# Morphology Evolution and Structural Reorganization of Enzyme–Biosurfactant Systems: Implications for Petroleum Interfacial Engineering

**DOI:** 10.3390/polym18182276

**Published:** 2026-09-17

**Authors:** Bo Huang, Han Cao, Wensen Zhao, Bo Jing, Yu Tian, Pengyao Xing, Dawei Wang

**Affiliations:** 1 China National Offshore Oil Corporation (CNOOC) Research Institute Ltd., Beijing 100028, China; ham_cao@163.com (H.C.); zhaows@cnooc.com.cn (W.Z.); jingbo@cnooc.com.cn (B.J.); tianyu12@cnooc.com.cn (Y.T.); wangdw3@cnooc.com.cn (D.W.); 2 State Key Laboratory of Offshore Oil and Gas Exploitation, Beijing 100028, China; 3 College of Chemistry and Chemical Engineering, Shandong University, Jinan 250100, China; xingpengyao@sdu.edu.cn

**Keywords:** biosurfactants, carbohydrate hydrolases, aggregation, topological morphology evolution

## Abstract

The escalating demand for sustainable chemical solutions in petroleum engineering has catalyzed the development of bio-based formulations characterized by high interfacial activity and low environmental toxicity. This study investigates the intermolecular interactions and structural evolution within composite systems comprising representative glycolipid biosurfactants and carbohydrate hydrolases. Rheological and light scattering assessments reveal that the introduction of enzymes triggers a transition from large-scale micelles to highly dispersed micro-aggregates, accompanied by a significant reduction in the system’s hydrodynamic resistance. Microscopic and spectroscopic evidence demonstrates that biosurfactants regulate enzyme aggregation behavior and promote the reorganization of amorphous enzyme clusters into more uniformly distributed microstructures through non-covalent interactions, including hydrogen bonding and hydrophobic interactions. Furthermore, time-resolved studies clarify a dynamic homogenization process where irregular aggregates evolve into stable, regular micro-aggregates. These findings provide fundamental insights into the structural organization and rheological behavior of enzyme–biosurfactant systems, offering a physicochemical foundation for the future rational design of bio-based formulations for potential petroleum-related interfacial engineering applications.

## 1. Introduction

The global petroleum industry is currently undergoing a fundamental paradigm shift driven by the dual necessity of meeting rising energy demands and adhering to increasingly stringent environmental regulations [1,2,3,4]. Traditional chemical enhanced oil recovery (EOR) and wellbore stimulation techniques have historically relied on synthetic surfactants such as alkylbenzene sulfonates and high-molecular-weight polymers like polyacrylamide [5,6,7]. While these reagents effectively reduce interfacial tension and control mobility, their ecological footprint remains a significant concern. The inherent toxicity and poor biodegradability of these synthetic compounds, along with their tendency to persist in groundwater and soil ecosystems, have catalyzed a movement toward green oilfield chemistry [8,9,10,11]. In this context, the development of bio-based formulations that exhibit high performance under extreme reservoir conditions is no longer merely an academic interest but an industrial imperative [10,12]. These conditions are typically defined by high salinity, elevated temperatures, and complex mineralogy, all of which pose severe challenges to the stability of conventional chemical agents [6,13].

Biosurfactants are amphiphilic secondary metabolites derived from microbial fermentation and have emerged as superior alternatives to conventional synthetic surfactants [14,15]. Among the most extensively documented classes are glycolipids, particularly sophorolipids (SLs) and rhamnolipids (RLs), alongside bio-derived nonionic surfactants such as alkyl polyglucosides (APGs) [16,17,18,19,20,21]. The structural diversity of these molecules provides a unique toolkit for interfacial engineering. Sophorolipids, which are produced by the yeast Candida bombicola, typically exist as a mixture of lactonic and acidic forms. The lactonic species is noted for its superior surface tension reduction, whereas the acidic form offers higher solubility in aqueous phases [18,22,23]. Rhamnolipids are primarily synthesized by Pseudomonas aeruginosa and contain one or two rhamnose units. These molecules possess an anionic character that facilitates strong interactions with positively charged rock surfaces or polyvalent cations [24,25]. In contrast, APGs offer a nonionic alternative with high tolerance to electrolyte concentrations. These biosurfactants are capable of reducing the surface tension of water and achieving ultralow oil–water interfacial tension, which is essential for mobilizing capillary-trapped residual oil. Furthermore, their ability to alter the wettability of carbonate or silicate reservoirs from oil-wet to water-wet states has been validated as a critical mechanism for improving recovery factors [6,26,27,28].

While the use of proteases and lipases is well-established in the detergent and food industries, the petroleum sector has recently turned its attention toward carbohydrate-active enzymes, also known as CAZymes [29,30,31,32,33]. In particular, amylase (Amy) and cellulase (Cel) play pivotal roles in managing the rheological properties of drilling fluids and the degradation of filter cakes [34,35]. During hydraulic fracturing and drilling operations, starch-based or cellulose-based polymers are frequently utilized as fluid-loss additives or thickening agents [36]. The accumulation of these polymeric residues can cause significant formation damage by restricting the flow of hydrocarbons toward the wellbore. Polysaccharide-degrading enzymes offer a highly specific and environmentally benign method for breaking these polymers into low-viscosity fragments, thereby restoring formation permeability. However, the efficacy of these enzymes is often compromised by the harsh physical conditions of the reservoir. Their tendency to form large aggregates in aqueous solutions may limit mass transfer and substrate accessibility at target interfaces [37,38,39].

Despite these advances, a critical analysis of the current literature reveals a significant disconnect between empirical observations and mechanistic understanding. A large body of existing research has predominantly focused on interactions between lipases, often characterized by their distinctive interfacial lid structures, and synthetic surfactants such as sodium dodecyl sulfate (SDS) [40,41,42,43,44]. These studies have established important principles concerning surfactant-induced protein conformational changes, denaturation, and interfacial activation. However, the mechanistic principles established for lipase–surfactant systems cannot be directly extrapolated to systems involving carbohydrate hydrolases, such as amylases and cellulases, and microbial or carbohydrate-based surfactants. Unlike lipases, carbohydrate hydrolases are generally globular and predominantly hydrophilic proteins whose catalytic function does not rely on the interfacial activation mechanism characteristic of many lipases. Consequently, the conventional emphasis on surfactant-induced unfolding may not fully capture the structural behavior of carbohydrate hydrolases in complex aqueous environments. Furthermore, much of the existing literature concerning biosurfactant-assisted bioremediation and enzyme formulations has employed crude microbial fermentation broths or complex multicomponent systems. Although such approaches are environmentally and technologically relevant, the presence of metabolic byproducts, residual nutrients, and other extracellular or cellular components can complicate the identification of direct structural interactions between enzymes and surfactants. To address this limitation, the present study employs a controlled binary system consisting of carbohydrate hydrolases and structurally distinct carbohydrate-based surfactants, including microbial glycolipid biosurfactants, namely sophorolipids (SLs) and rhamnolipids (RLs), together with the nonionic alkyl polyglucoside (APG) [16,17,18,19,20,21]. The selection of these surfactants provides a comparative platform for examining how differences in hydrophilic headgroup architecture, charge characteristics, and amphiphilicity influence enzyme aggregation and structural organization.

We hypothesize that the carbohydrate-containing headgroups and amphiphilic architectures of these surfactants may facilitate favorable non-covalent interactions with carbohydrate hydrolases, including hydrogen bonding and hydrophobic association, rather than necessarily inducing the extensive protein-denaturing effects commonly observed with strongly interacting synthetic surfactants [43,44,45]. Such interactions may not only influence the molecular conformation of individual enzymes but may also regulate their higher-order association and aggregation behavior. Previous work has shown that rhamnolipid can interact with industrial cellulase and α-amylase without substantially compromising their structural integrity or catalytic activity under the investigated conditions [46]. However, the higher-order aggregation and morphological reorganization of carbohydrate hydrolase aggregates induced by different biosurfactant architectures remain insufficiently understood [46,47]. By deconstructing the complex enzyme–biosurfactant interface into a controlled binary model, this study integrates hydrodynamic, microscopic, spectroscopic, rheological, and enzymatic activity analyses to characterize the system across multiple length scales. It seeks to establish a multiscale framework for understanding biosurfactant-mediated structural reorganization and its potential relevance to the rational design of enzyme-containing formulations for petroleum interfacial engineering.

The findings presented herein highlight that surfactant-induced disassembly of enzyme clusters represents a key structural mechanism influencing composite behavior. By revealing how microstructural organization governs temperature-dependent viscosity and dispersion characteristics, this study advances the fundamental understanding of enzyme–biosurfactant hybrid systems and provides a physicochemical basis for their further evaluation in petroleum-related interfacial engineering.

## 2. Materials and Methods

All biosurfactants (RLs (95%), SLs (>99%), and APGs (>98%)) were purchased from Merck. Amylase (Amy) and cellulase (Cel) were produced in-house by fermentation using *Priestia megaterium* and *Geobacillus* sp. 70PC53, respectively. The obtained enzyme preparations were subsequently collected and processed according to the procedures described in the Supplementary Materials. The protein content and enzymatic activities of Amy and Cel are provided in the Supplementary Materials.

The sample preparation procedure was as follows: aqueous solutions of surfactants and enzyme preparations were first prepared separately at a concentration of 100 g/L. The two solutions were then mixed at a 1:1 (*v*/*v*) ratio and sonicated for 60 min at a power of 100 W to ensure homogeneous dispersion, generating the surfactant-enzyme composite systems. All solutions were maintained under neutral pH conditions and aged for 24 h before characterization. Detailed information regarding the enzymatic activities of the enzyme preparations and the compositions of the surfactants is provided in the Supplementary Materials.

Dynamic light scattering (DLS) measurements were performed using a Zetasizer Pro instrument (Malvern Panalytical, Malvren, UK) to determine the hydrodynamic diameter and size distribution of the samples. All measurements were conducted at 25 °C using a backscattering detection mode (173° scattering angle) with a 633 nm laser. Before each measurement, samples were equilibrated for 10 min. The hydrodynamic diameter (Z-average) and polydispersity index (PDI) were obtained from cumulant analysis. Each sample was measured at least five times, and the averaged values were reported to ensure reproducibility.

For SEM measurements, the aqueous sample solutions were diluted to the desired concentration and deposited onto silicon substrates. The samples were dried at room temperature to remove the solvent, followed by sputter coating with a thin gold layer before imaging. SEM observations were performed using a field-emission scanning electron microscope with an accelerating voltage of 3.00 kV.

The nanostructural features of the assembled samples were investigated by transmission electron microscopy (TEM). The aqueous dispersions were diluted to a suitable concentration, and aliquots of the samples were dropped onto carbon-coated copper grids. After incubation for 3 min, the excess solution was removed with filter paper. TEM images were collected using a transmission electron microscope (HITACHI JEM-100CX II) at an accelerating voltage of 100.0 kV.

The viscosity was measured using a rotational rheometer at 25 °C under controlled shear rate mode. The shear rate was logarithmically increased from 0.1 to 100 s^−1^, and the apparent viscosity was calculated from the measured shear stress.

Different concentrations were selected according to the requirements of individual characterization techniques. Samples at 100 g/L were used for rheological measurements and concentrated-state SEM observations to evaluate the macroscopic aggregation and flow behaviors, whereas samples at 10 g/L were employed for DLS and TEM measurements to minimize concentration-induced scattering interference and facilitate nanoscale structural characterization.

## 3. Results and Discussion

**Figure 1:**
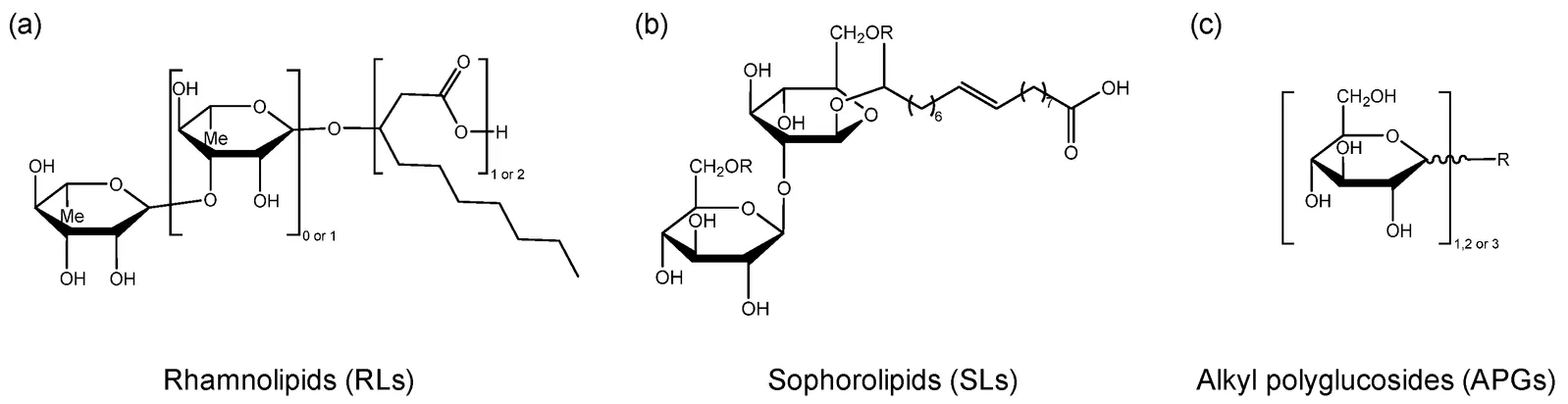
Molecular structures of the biosurfactants used in this study: (**a**) Rhamnolipids (RLs), (**b**) Sophorolipids (SLs) and (**c**) Alkyl polyglucosides (APGs).

Biosurfactants and sugar-based surfactants have attracted increasing attention in petroleum related applications because of their excellent interfacial activity, environmental compatibility, and ability to regulate multiphase systems. In oilfield processes such as enhanced oil recovery, crude oil demulsification, and oil–water separation, these surfactants are frequently introduced to modify interfacial properties and to regulate the stability and microstructure of dispersed systems. Among the various candidates, sophorolipids (SLs), rhamnolipids (RLs), and alkyl polyglucosides (APGs) represent three typical surfactants that have been widely investigated and applied due to their high surface activity, biodegradability, and good compatibility with biological systems (Figure 1). Structurally, SLs consist of a sophorose disaccharide linked to a hydroxy fatty acid and can exist in either lactonic or acidic forms. RLs contain one or two rhamnose units connected to β-hydroxy fatty acids and usually exhibit an anionic character in aqueous solution. In contrast, APGs are nonionic surfactants formed by the glycosidic linkage between glucose units and long-chain fatty alcohols. Although these surfactants share a common amphiphilic framework composed of carbohydrate headgroups and hydrophobic alkyl chains, they differ significantly in headgroup structure and molecular charge. The selection of these three surfactants therefore provides a representative and structurally diverse system that allows a systematic investigation of how different biosurfactant architectures influence the interaction with enzymes and the resulting assembly behavior in aqueous environments.

Building upon the introduction of the selected biosurfactants, the enzymatic component of the system also warrants consideration. Compared with proteases and lipases that have been more widely explored in oilfield related enzymatic processes, polysaccharide degrading enzymes have received relatively limited attention, despite their potential to regulate carbohydrate rich structures and complex organic aggregates. Among this class of enzymes, amylase (Amy) and cellulase (Cel) represent two representative carbohydrate hydrolases with distinct catalytic characteristics.

**Figure 2:**
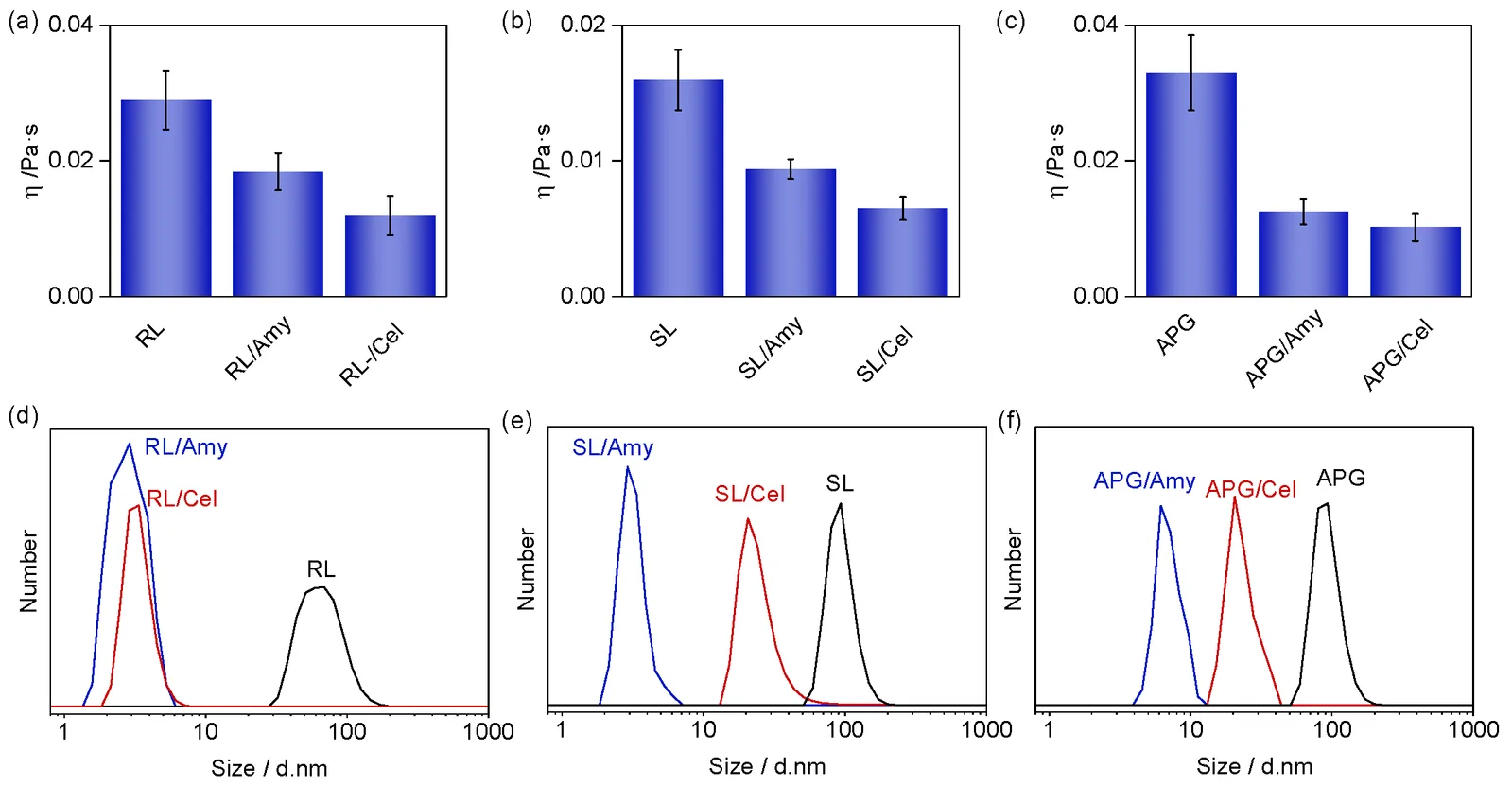
(**a**) Viscosity comparison of RL, RL/Amy, and RL/Cel. (**b**) Viscosity comparison of SL, SL/Amy, and SL/Cel. (**c**) Viscosity comparison of APG, APG/Amy, and APG/Cel. (**d**) Dynamic light scattering (DLS) size distributions of RL, RL/Amy, and RL/Cel. (**e**) DLS size distributions of SL, SL/Amy, and SL/Cel. (**f**) DLS size distributions of APG, APG/Amy, and APG/Cel. The samples used for the viscosity measurements were aqueous solutions with a concentration of 100 g/L after aging for 24 h. The samples used for DLS measurements were aqueous solutions with a concentration of 10 g/L after aging for 24 h.

To investigate the interactions between the selected enzymes and surfactants, the apparent viscosity of the mixed systems was first examined. As a macroscopic parameter sensitive to changes in molecular organization and aggregation behavior, apparent viscosity provides preliminary insight into the influence of enzyme-surfactant interactions on the physicochemical properties of the systems (Figure 2a–c). First, the samples were prepared as aqueous solutions with a concentration of 100 g/L and then aged for 24 h. The apparent viscosity of each sample was subsequently measured at room temperature using a rheometer. The apparent viscosities of the three surfactants were all on the order of 10^−2^ Pa·s. Upon the addition of the enzyme, the apparent viscosity of the samples decreases slightly, but remains on the order of 10^−2^. A decrease in apparent viscosity generally reflects a reduction in the effective hydrodynamic volume and structural connectivity of the dispersed phase, which may arise from the disruption of highly interconnected aggregates, the formation of smaller aggregates, or the transition toward a more loosely organized microstructure [45,48,49]. In aqueous media, biosurfactants can spontaneously self-assemble into micelles and other supramolecular aggregates [50], and the resulting aggregate size, morphology, hydration state, and interparticle interactions collectively contribute to the flow resistance of the system. The incorporation of enzyme molecules may perturb the self-assembly equilibrium of the biosurfactants through non-covalent interactions [51], thereby altering the organization and connectivity of the surfactant aggregates. Conversely, adsorption or association of biosurfactant molecules with enzyme aggregates may weaken enzyme-enzyme interactions and facilitate the reorganization of highly associated structures into smaller and less interconnected aggregates. Such structural reorganization can reduce the effective hydrodynamic volume and the formation of transient networks within the dispersion, ultimately lowering the resistance to flow and thus the apparent viscosity.

Therefore, dynamic light scattering analysis (DLS) was performed to obtain the particle size distribution of the aggregates in the system (Figure 2d–f). The samples were prepared as dilute aqueous solutions with a concentration of 10 g/L and aged for 24 h. Dynamic light scattering measurements were then performed at room temperature using a nanoparticle size analyzer. Generally, surfactants form micelles of various sizes in aqueous solution. The number-distribution peak diameters of the three surfactants fall within the range of 50–100 nm. A dominant scattering population with a hydrodynamic size of approximately 3 nm emerges in RL/Amy and RL/Cel mixtures after enzyme addition. It should be noted that ~3 nm is relatively small for aggregates containing enzyme molecules. We cannot completely exclude the possibility that part of this signal originates from free surfactant monomers or small surfactant oligomers. Given the binary nature of our mixed system, DLS alone cannot definitively assign this population exclusively to enzyme-biosurfactant aggregates. Additional complementary experiments such as size-exclusion chromatography or fluorescence labelling would be needed to unambiguously assign the origin of this scattering population in future work. The reduction in apparent viscosity is consistent with the DLS results showing a decrease in micelle size after enzyme addition. This may suggest that the disruption or reorganization of surfactant aggregates leads to smaller aggregates with lower hydrodynamic resistance [52].

Notably, the three surfactant–enzyme pairs exhibit divergent extents of size reduction upon enzyme addition, which can be related to their distinct molecular architectures. RL and SL are anionic glycolipid biosurfactants bearing ionizable carboxylate head-groups, enabling electrostatic interactions with charged amino-acid residues on enzyme surfaces. In contrast, APG is a nonionic alkyl polyglucoside without ionizable charged groups; its intermolecular interactions with enzymes are dominated by hydrophobic contacts and hydrogen bonding. Such differences in dominant interaction modes contribute to the varied aggregate reorganization behaviour observed for RL/enzyme, SL/enzyme and APG/enzyme systems.

It should also be noted that DLS measurements were performed at 10 g/L. At this relatively high concentration, inter-particle interactions and potential multiple-scattering effects may influence the measured size distribution. Therefore, the obtained results reflect the assembly behavior under this specific experimental concentration, and may not be directly extrapolated to highly diluted conditions.

It should be noted that the variation in apparent viscosity does not completely follow the trend of hydrodynamic size obtained from DLS. This discrepancy arises because viscosity reflects the collective flow behavior of the whole system, while DLS mainly provides information on the size distribution of scattering species. In addition to aggregate size, factors including aggregate morphology, interparticle interactions, and structural connectivity strongly influence the rheological response. Therefore, the lower viscosity observed for SL/Cel may be associated with the reduced intermolecular interactions and weaker structural connectivity within the composite system, rather than simply a decrease in aggregate size.

**Figure 3:**
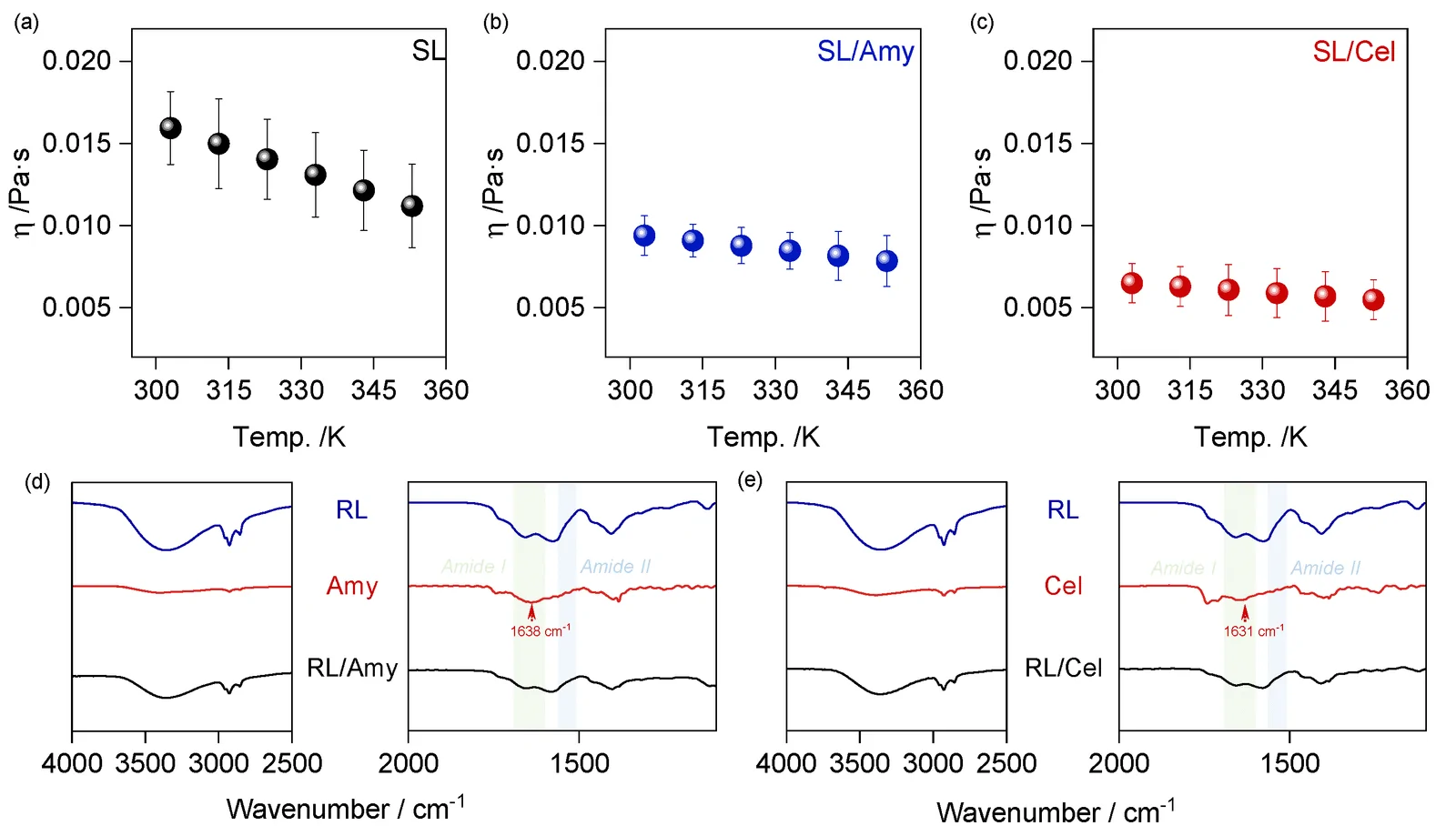
Temperature-dependent viscosity of SL (**a**), SL/Amy (**b**), and SL/Cel (**c**). (**d**) Fourier transform infrared (FTIR) spectra of RL, Amy, and RL/Amy. (**e**) Fourier transform infrared (FTIR) spectra of RL, Cel, and RL/Cel. The samples used for the apparent viscosity measurements were aqueous solutions with a concentration of 100 g/L after aging for 24 h.

To further understand the evolution of aggregate behavior in the system, the apparent viscosity was also measured at different temperatures (Figure 3a–c, Figures S1 and S2). The samples were prepared following the same procedure as that used for the apparent viscosity measurements described above. During testing, each sample was equilibrated at the target temperature for 10 min before measurement. At each temperature, the measurement was repeated 20 times. The temperature was gradually increased from 303 K to 353 K. The apparent viscosity of SL alone shows a decreasing trend with increasing temperature. This may be associated with the enhanced thermal motion of molecules at elevated temperatures, which may affect hydrophobic interactions, hydrogen bonding, and van der Waals interactions among surfactant molecules [43,53]. As a result, the original micellar or aggregated structures become more loosely organized, the internal frictional resistance of the system decreases, and the apparent viscosity of the SL solution correspondingly declines. After the addition of enzymes, the apparent viscosity of the system still decreases with increasing temperature, but the magnitude of the decrease is smaller than that observed for SL alone. This behavior may arise because, upon the introduction of enzymes, amino acid residues on the enzyme surface can interact with SL molecules through hydrogen bonding, electrostatic interactions, or hydrophobic interactions [53,54], thereby forming partially associated complexes or more stable aggregates. These interactions may modify intermolecular associations within SL-enzyme mixtures and give rise to a less-pronounced viscosity decrease upon heating relative to pure SL. Nevertheless, this macroscopic rheological behaviour alone cannot provide definitive evidence for improved thermal stability of enzyme-biosurfactant aggregates. Multiple intertwined factors, including molecular thermal motion, hydrogen-bonding, hydrophobic and inter-particle interactions, collectively determine the temperature-dependent viscosity response.

Meanwhile, the particle sizes of the enzymes themselves were compared with those of the aggregates. After the addition of the surfactant, the particle size of the enzyme also showed a significant decrease. This observation suggests that the surfactant may disrupt the original aggregation of the enzyme and lead to the formation of smaller aggregates that are more uniformly dispersed within the surfactant system. To further understand the structural characteristics of the aggregates, Fourier transform infrared (FTIR) spectroscopy was performed (Figure 3d,e, Figures S3 and S4). The FTIR spectrum of the composite system retains the characteristic absorption bands of both individual components on the whole, although changes in band position and intensity can be observed in several spectral regions dominated by the surfactant. These results indicate that the characteristic chemical skeletons of the individual components are largely retained after mixing, while the local molecular environments may be altered upon biosurfactant–enzyme association.

For pure Amy and Cel samples, the amide I bands are centered at 1638 cm^−1^ and 1631 cm^−1^, respectively. The theoretically expected amide II absorption within the 1510–1560 cm^−1^ region overlaps heavily with the broad tail of the amide I band and cannot be resolved as a separate distinct peak in both pure enzyme spectra. In composite systems, the amide-I signals originating from the enzyme component are completely undetectable due to the low enzyme content, so reliable analysis of peak-position shifts or intensity variations of enzyme amide bands cannot be performed. Without band-fitting or deconvolution analysis, the present FTIR data cannot provide quantitative information about protein secondary-structure rearrangement, nor can they unambiguously identify specific intermolecular forces such as hydrogen-bonding or hydrophobic interactions. The overall spectral changes observed are merely consistent with the occurrence of non-covalent biosurfactant-enzyme interactions as a plausible scenario. From the FTIR perspective, the fundamental chemical features of the system are not altered; instead, the system evolves from larger-sized aggregates toward smaller aggregates while preserving the main infrared signatures of the individual components.

To determine whether the structural reorganization induced by biosurfactants affects enzyme functionality, the enzymatic activities of amylase and cellulase before and after interaction with different biosurfactants were evaluated. As shown in Table S1, both Amy and Cel retained high relative activities after incubation with SL, RL, and APG, with no significant loss of catalytic activity compared with the corresponding enzyme-only systems. These results indicate that the biosurfactant-induced dispersion of enzyme aggregates does not significantly impair the catalytic activity of the enzymes. Therefore, the observed reduction in aggregate size is accompanied by the preservation of enzymatic function rather than apparent enzyme deactivation.

The morphology of the enzyme-surfactant aggregates was further characterized by transmission electron microscopy (TEM). The samples were prepared under the same conditions as those used for the DLS measurements. Briefly, the samples were deposited onto copper grids and naturally dried before TEM observation. It should be noted that the drying process during TEM sample preparation may induce partial structural rearrangement; therefore, the TEM images were interpreted as comparative morphological evidence rather than direct representations of the hydrated structures in solution. Accordingly, all electron-microscopy results in this work are strictly qualitative and serve only for comparative morphological assessment between samples. Quantitative particle-size analysis is not performed based on TEM or SEM images.

**Figure 4:**
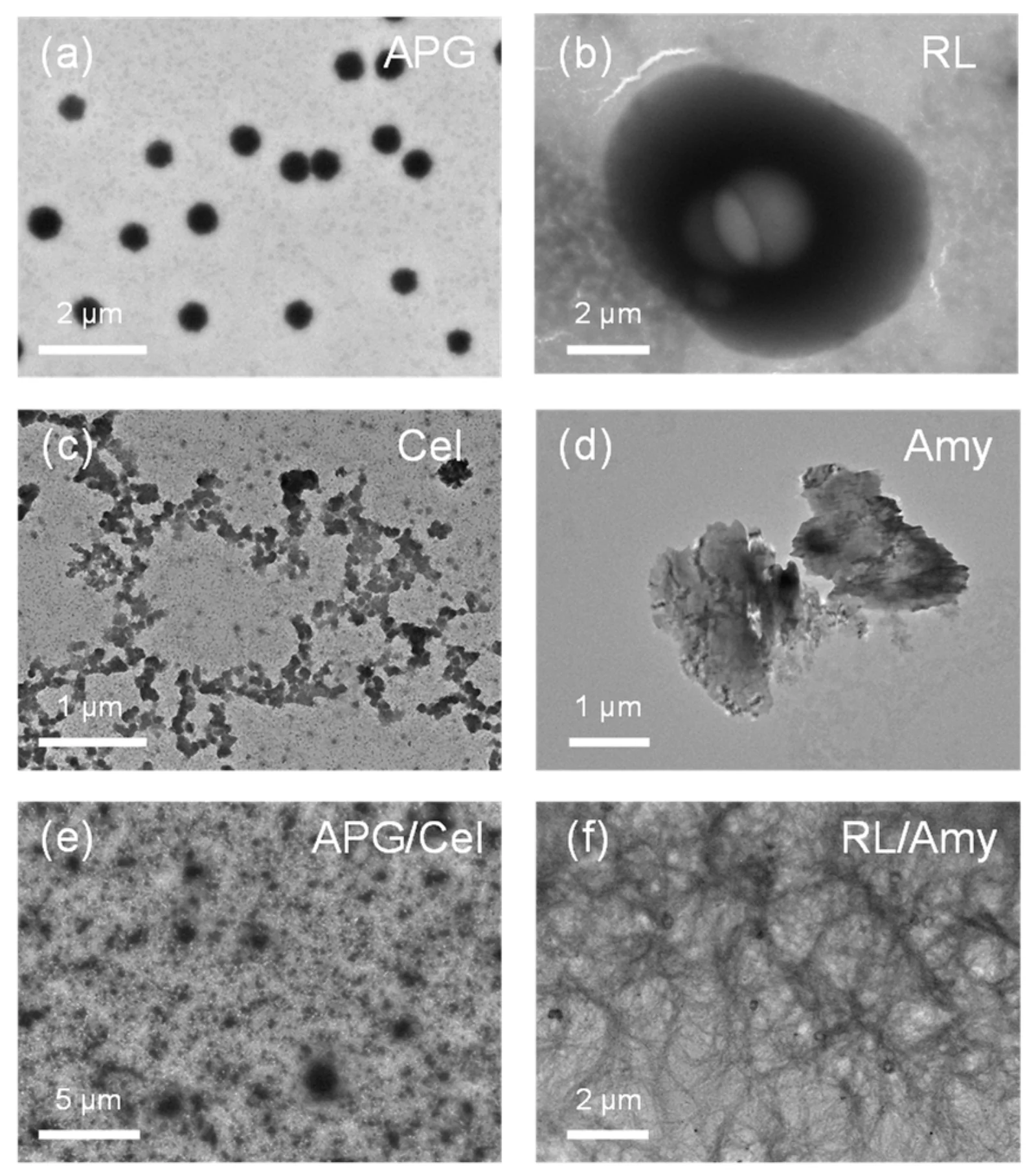
Transmission electron microscopy (TEM) images of (**a**) APG, (**b**) RL, (**c**) Cel, (**d**) Amy, (**e**) APG/Cel, and (**f**) RL/Amy. The samples were aqueous solutions with a concentration of 10 g/L after aging for 24 h.

The dried surfactant samples displayed spherical features (Figure 4a,b and Figure S5). Among them, APG and SL showed relatively compact spherical structures, whereas RL exhibited hollow-like spherical features with internal contrast variations. In contrast, the dried enzyme samples (Cel and Amy) exhibited irregular amorphous morphologies (Figure 4c,d). After incorporation of surfactants, the enzyme-containing samples showed more dispersed and organized morphological features. For example, APG/Cel exhibited relatively uniform spherical features, whereas RL/Amy displayed fibrous-like structures (Figure 4e,f). Similar morphological differences were also observed by scanning electron microscopy (SEM, Figures S7 and S8). These observations are consistent with the size variation trends obtained from DLS and the interaction changes indicated by FTIR analysis.

**Figure 5:**
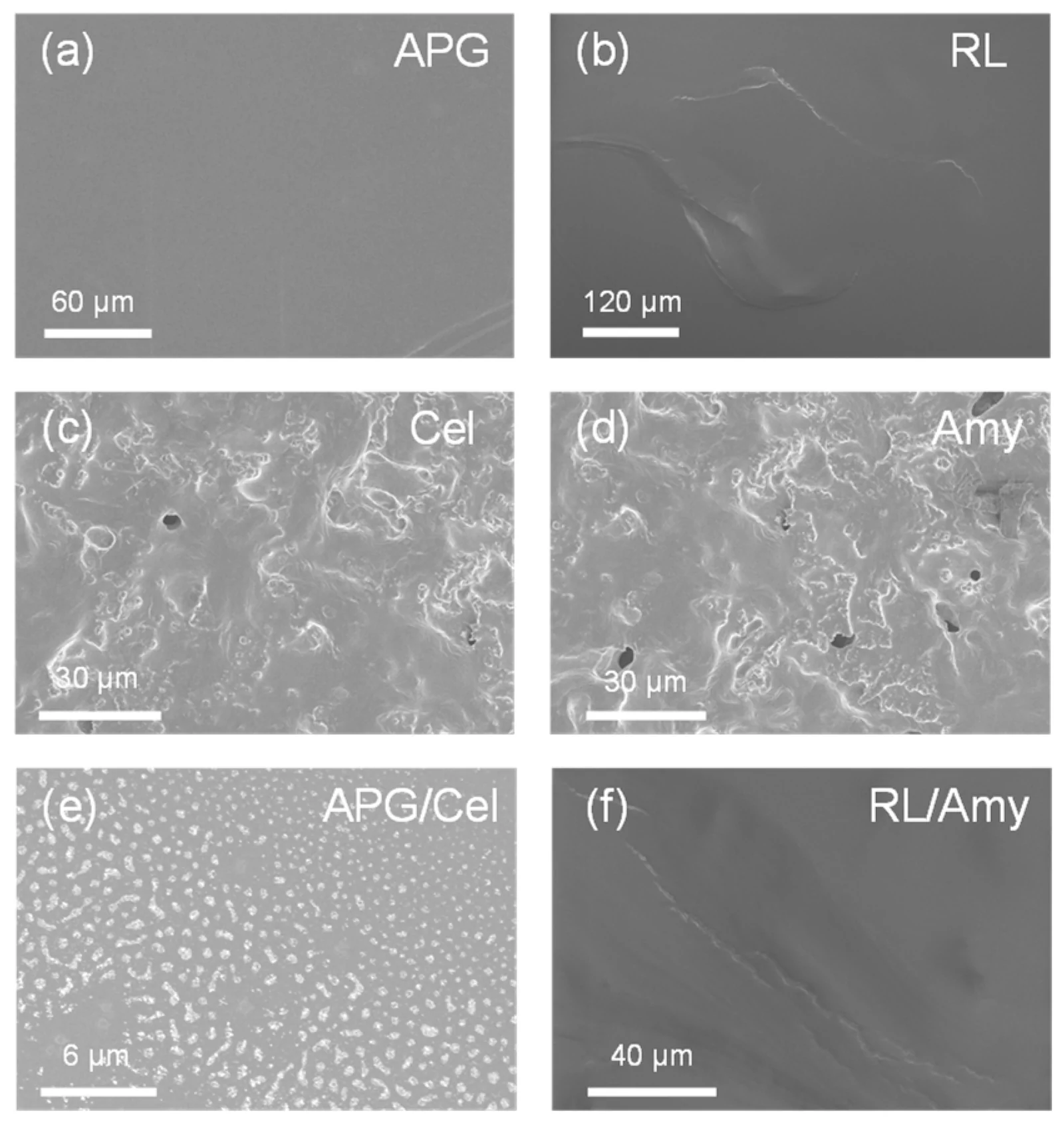
Scanning electron microscopy (SEM) images of (**a**) APG, (**b**) RL, (**c**) Cel, (**d**) Amy, (**e**) APG/Cel, and (**f**) RL/Amy. The samples were aqueous solutions with a concentration of 100 g/L after aging for 24 h.

To further investigate the morphological characteristics under relatively concentrated conditions relevant to practical oilfield applications, the concentrations of surfactants and enzymes were increased. The samples were uniformly deposited onto silicon wafers to prepare dried thin films, and the surface morphologies were examined by SEM (Figure 5).

The films prepared from surfactants alone exhibited relatively smooth surfaces, whereas the enzyme-containing films showed irregular and rough morphologies. Similar morphological differences to those observed by TEM were observed. The surfaces of APG/Cel and RL/Amy composite films retained relatively rough features associated with the enzyme components. However, after surfactant incorporation, the film surfaces became relatively more homogeneous. These results suggest that surfactant addition modifies the aggregation morphology of enzyme-containing systems and promotes the formation of more uniformly distributed dried-state structures.

**Figure 6:**
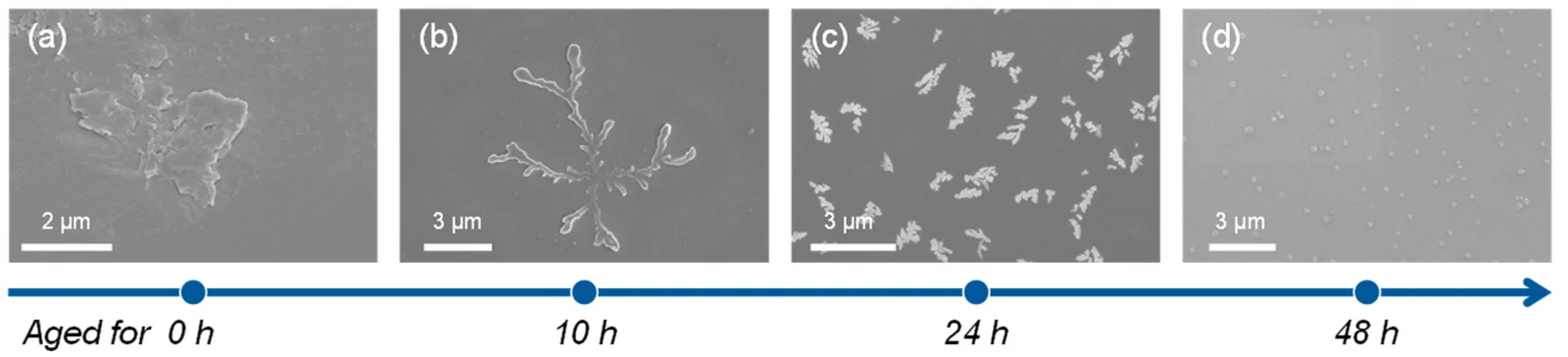
(**a**–**d**) Time-dependent scanning electron microscopy (SEM) images of APG/Cel samples prepared after aging for 0, 10, 24, and 48 h. The samples were aqueous solutions with a concentration of 10 g/L after aging for different time.

In addition, the evolution of the microscopic topological morphology of the composite aggregates with aging time was investigated. APG/Cel was selected as the representative system and prepared as a dilute aqueous solution with a concentration of 10 g/L. SEM images were recorded after aging for 0, 10, 24, and 48 h. At 0 h stage (Figure 6a), APG/Cel exhibits an amorphous block-like morphology, which is similar to that of the enzyme itself. This observation indicates that, at this stage, the surfactant has not yet dispersed the large enzyme aggregates into smaller ones, and the microscopic morphology remains similar to that of the native enzyme aggregates. At the 10 h stage (Figure 6b), the aggregates evolve into a dendritic morphology. However, the terminal regions still exhibit smooth and irregular features. The overall structure shows a radial appearance, suggesting that the enzyme aggregates have begun to fragment and are likely to further evolve into smaller aggregates. At the 24 h stage (Figure 6c), the transformation of the enzyme aggregates from large aggregates to smaller ones is essentially completed. At this point, the composite system exhibits a morphology consisting of uniformly distributed block-like structures. This morphology is similar to that observed at 0 h, but the structures at 24 h are relatively smaller and more regular. At the 48 h stage (Figure 6d), the morphology of the composite system undergoes further evolution, forming even smaller structures. This time-resolved SEM evolution clearly illustrates the process by which the surfactant disperses large enzyme aggregates into smaller aggregates.

## 4. Conclusions

In summary, this study systematically investigated the interactions and assembly behaviors between representative biosurfactants (SLs, RLs, APGs) and enzymes (Amy, Cel) in aqueous systems. Viscosity and DLS analyses showed that the incorporation of enzymes was accompanied by changes in the hydrodynamic size and apparent viscosity of the biosurfactant systems, while the presence of biosurfactants was associated with a decrease in the apparent size and morphological heterogeneity of enzyme-containing aggregates. FTIR spectroscopy and temperature-dependent viscosity measurements demonstrated that these structural transitions are driven by non-covalent interactions. These observations are consistent with the occurrence of non-covalent interactions between biosurfactants and enzymes, although the specific molecular contributions of individual interactions cannot be directly established from the present experiments. Time-resolved SEM observations further showed that the morphology of the APG/Cel system evolved with aging time, from irregular and block-like structures toward smaller and more uniformly distributed structures under the investigated conditions. Collectively, the experimental results demonstrate that biosurfactant incorporation is associated with changes in the aggregation state, hydrodynamic behavior, and microscopic morphology of enzyme-containing systems. These findings provide a physicochemical basis for understanding biosurfactant-enzyme assembly behavior and may inform the development of enzyme–biosurfactant formulations for potential petroleum-related applications. Further studies are required to evaluate the structural stability and interfacial performance of these complexes under representative petroleum-related conditions (e.g., oil–water interface, mineral surfaces, high salinity and elevated temperature), including their applicability to enhanced oil recovery and crude oil demulsification.

## Data Availability

The original contributions presented in this study are included in the article/Supplementary Material. Further inquiries can be directed to the corresponding authors.

## Supplementary Materials

The following supporting information can be downloaded at: https://www.mdpi.com/article/10.3390/polym18182276/s1, Table S1: Results of viscosity-reduction activity assay. Table S2: Hydrodynamic diameter, polydispersity index, and size distribution parameters of enzyme–biosurfactant assemblies determined by dynamic light scattering (DLS). Figure S1: Temperature-dependent viscosity of APG, APG/Amy, and APG/Cel. Figure S2: Temperature-dependent viscosity of RL, RL/Amy, and RL/Cel. Figure S3: Fourier transform infrared (FTIR) spectra of APG, Amy, and APG/Amy. And Fourier transform infrared (FTIR) spectra of APG, Cel, and APG/Cel. Figure S4: Fourier transform infrared (FTIR) spectra of SL, Amy, and SL/Amy. And Fourier transform infrared (FTIR) spectra of SL, Cel, and SL/Cel. Figure S5: Transmission electron microscopy (TEM) images of SL, SL/Cel, and SL/Amy. Figure S6: Transmission electron microscopy (TEM) images of APG/Amy, and RL/Cel. Figure S7: Scanning electron microscopy (SEM) images of Cel, Amy, APG, SL, and RL. Figure S8. Scanning electron microscopy (SEM) images of APG/Cel, APG/Amy, SL/Cel, SL/Amy, RL/Cel, and RL/Amy. Figure S9: Scanning electron microscopy (SEM) images of SL, SL/Cel, and SL/Amy. The samples were aqueous solutions with a concentration of 100 g/L after aging for 24 h. Figure S10: Scanning electron microscopy (SEM) images of APG/Amy, and RL/Cel. The samples were aqueous solutions with a concentration of 100 g/L after aging for 24 h.
