# Supplementary material for: Morphology Evolution and Structural Reorganization of Enzyme–Biosurfactant Systems: Implications for Petroleum Interfacial Engineering
Source: Polymers (Basel). 2026 Sep 17;18(18):2276. doi: 10.3390/polym18182276 (PMC13611511; doi:10.3390/polym18182276)
Supplement: Supplementary file 1 [file polymers-18-02276-s001.zip › polymers-4547017-supplementary.pdf]

Supporting Information

Content

A. Experimental Procedures S1

B. Additional experimental data..S3

A.Experimental Procedures

Biosurfactants.

All biosurfactants (RLs (95%), SLs (>99%), and APGs (>98%)) were purchased from Merck.

Protein content of the enzyme preparation

First, the cells were collected by centrifugation and subsequently disrupted by sonication. Therefore, the protein concentrations of the supernatant after cell lysis were all determined (Amy: 40~50 kDa, 0.547 mg/L; Cel: 40~50 kDa, 0.634 mg/L).

Viscosity-reduction assay for Amy and Cel

The viscosity-reduction assay was used to evaluate the hydrolytic activity of Amy and Cel. Sodium carboxymethyl cellulose (CMC-Na) served as the substrate for Cel activity measurement, while soluble starch was used as the substrate for Amy. All reaction systems were buffered to pH 7.0 with a salinity of 2 wt%, and reactions were carried out at 35 °C.

Substrate stock solutions were prepared in pH 7.0 buffer containing 2 wt% salt. For each reaction, 9.0 mL substrate solution was pre-incubated at 35 °C for 15 min. Subsequently, 1.0 mL diluted crude enzyme sample (100g/L) was added and mixed thoroughly to start the enzymatic reaction, giving a total reaction volume of 10.0 mL.

A blank control was run in parallel, where an equal volume of heat-inactivated enzyme (boiled for 10 min) replaced the active enzyme solution, with all other incubation conditions kept identical.

Upon completion of the scheduled incubation period, reaction mixtures were rapidly cooled to stop polysaccharide hydrolysis. The viscosities of both sample and blank control solutions were measured by viscometer at 35 °C.

The viscosity-reduction rate, defined by the viscosity difference between blank control and enzyme-treated sample, was adopted to reflect relative enzyme activity. A higher viscosity-reduction rate corresponds to stronger polysaccharide-degrading capability. All assays were performed in triplicate.

Table S1 Results of viscosity-reduction activity assay

Entry	Activity
Amy	89.9%
Amy/RL	92.3%
Amy/SL	99.6%
Amy/APG	92.6%
Cel	88.7%
Cel/RL	97.8%
Cel/SL	72.2%
Cel/APG	99.6%

Table S2. Hydrodynamic diameter, polydispersity index, and size distribution parameters of enzyme–biosurfactant assemblies determined by dynamic light scattering (DLS)

Sample	Z-average diameter (nm)	PDI	Number peak diameter (nm)
SL/Amy	3.8	0.21	2.9
SL/Cel	30.2	0.29	20.5
RL/Amy	4.1	0.22	2.9
RL/Cel	4.8	0.20	3.4
APG/Amy	9.5	0.25	6.1
APG/Cel	31.5	0.31	20.5
RL	95.0	0.37	68.7
SL	125.0	0.42	92.9
APG	130.0	0.40	92.9

Amino acid sequences of enzymes used in this study.

Amy:

MLTFHRIIRKGWMFLLAFLLTASLFCPTGQPAKAAPPFNGTMMQYFEWYLPD
DGTLWTKVANEANNLSSLGITALWLPPAYKGTSRSDVGYGVYDLYDLGEFNQ
KGTVRTKYGTKAQYLQAIQAAHAAGMQVYADVVFDHKGGADGTEWVDAV
EVNPSDRNQEISGTYQIQAWTKFDFPGRGNTYSSFKWRWYHFDGVDWDESR

KLSRIYKFRGIGKAWDWEVDTENGNYDYLMYADLDMDHPEVVTELKNWGK

WYVNTTNIDGFRLDAVKHIKFSFFPDWLSYVRSQTGKPLFTVGEYWSYDINK

LHNYITKTNGTMSLFDAPLHNKFYTASKSGGAFDMRTLMTNTLMKDQPTLA

VTFVDNHDTEPGQALQSWVDPWFKPLAYAFILTRQEGYPCVFYGDYYGIPQY

NIPSLKSKIDPLLIARRDYAYGTQHDYLDHSDIIGWTREGVTEKPGSGLAALIT

DGPGGSKWMYVGKQHAGKVFYDLTGNRSDTVTINSDGWGEFKVNGGSVSV

WVPRKTTVSTIAWPITTRPWTGEFVRWTEPRLVAWP

Cel:

MERTPVEENGRLQVVGTALLNQHNKPFQLRGISTHGLQWFGQFANKDAFQTL

RDDWKANVVRLAMYTDPNANGYIAQPEWLKAKVKEGVQAALDLGMYVIID

WHILNDNDPNLYKEQAKRFFAEMAREYGKYPNVIYEIANEPNGNDVTWEEKI

RPYADEVIRTIRSIDRDNLIIVGTGTWSQDVDDVASDPLPYKNIMYAVHFYSGT

HTQWLRDRVDAALQAGTPVFVSEWGTSDASGDGGPYLEEAEKWIEFLNERGI

SWVNWSLCDKNEASAALRPGADPHGGWGDDHLSDSGRFIKAKLIEALE

B. Additional experimental data

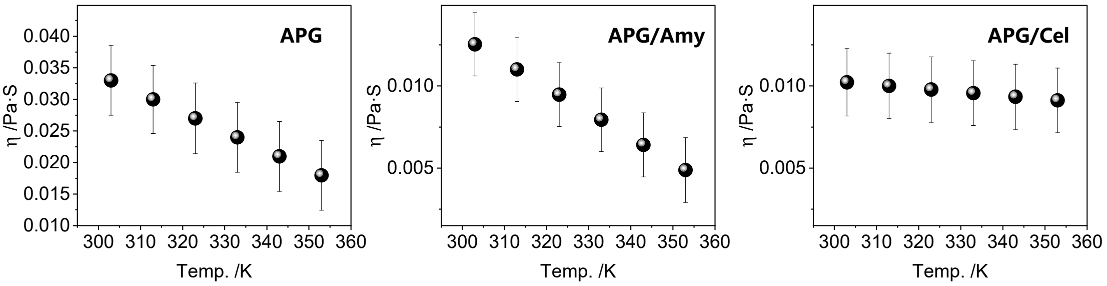

Figure S1. Temperature-dependent viscosity of APG, APG/Amy, and APG/Cel.

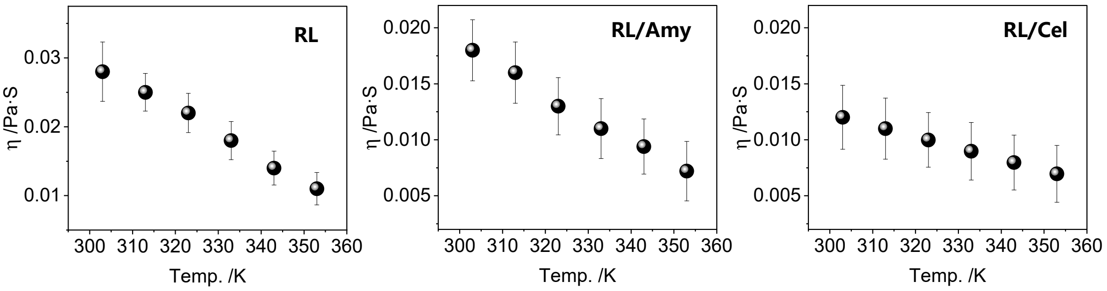

Figure S2. Temperature-dependent viscosity of RL, RL /Amy, and RL /Cel.

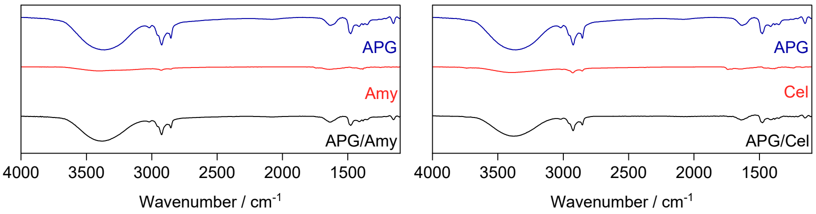

Figure S3. Fourier transform infrared (FTIR) spectra of APG, Amy, and APG /Amy. And Fourier transform infrared (FTIR) spectra of APG, Cel, and APG /Cel.

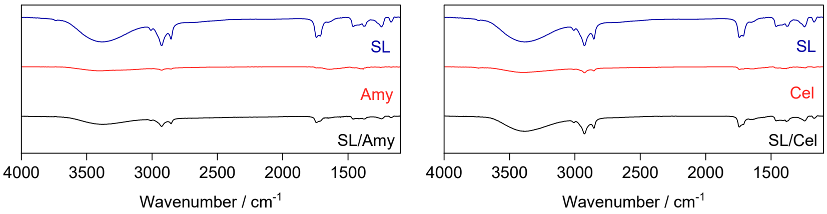

Figure S4. Fourier transform infrared (FTIR) spectra of SL, Amy, and SL /Amy. And Fourier transform infrared (FTIR) spectra of SL, Cel, and SL /Cel.

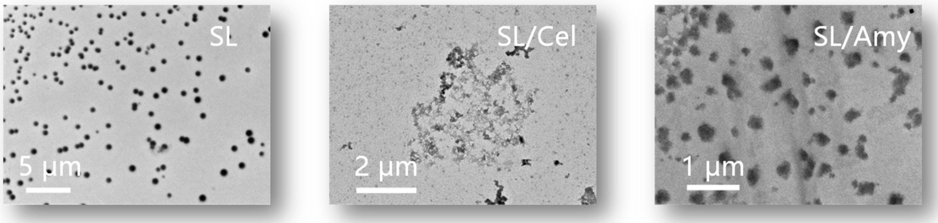

Figure S5. Transmission electron microscopy (TEM) images of SL, SL/Cel, and SL /Amy.

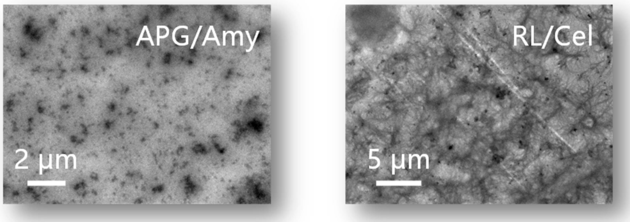

Figure S6. Transmission electron microscopy (TEM) images of APG/Amy, and RL/Cel.

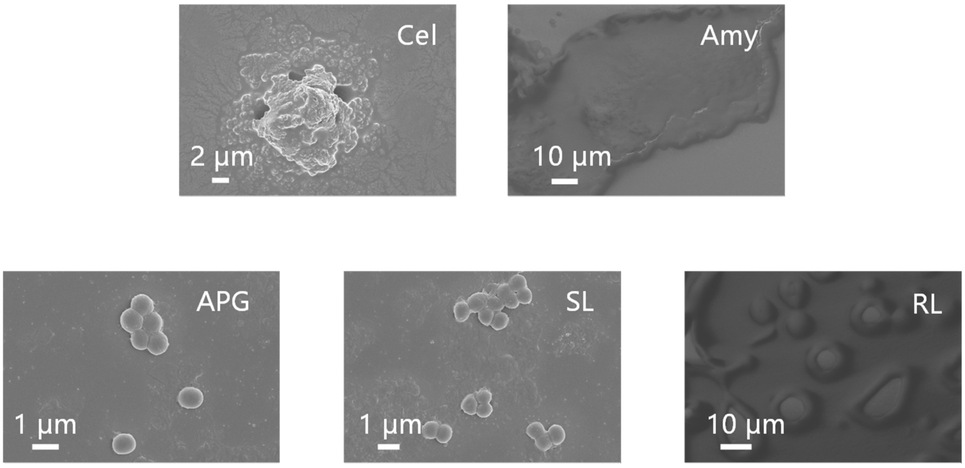

Figure S7. Scanning electron microscopy (SEM) images of Cel, Amy, APG, SL, and RL.

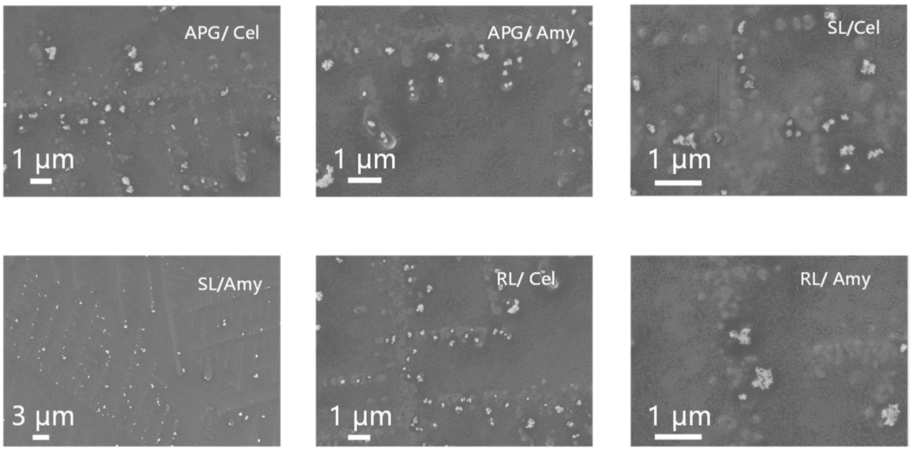

Figure S8. Scanning electron microscopy (SEM) images of APG/Cel, APG/Amy, SL/Cel, SL/Amy, RL/Cel, and RL/Amy.

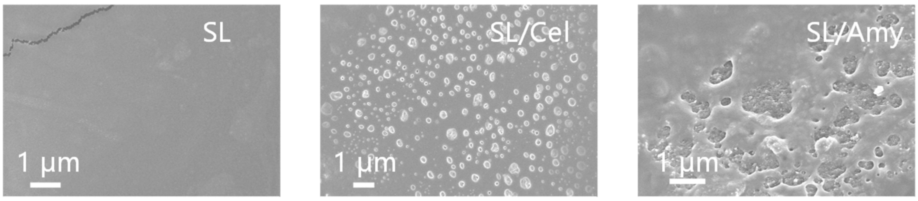

Figure S9. Scanning electron microscopy (SEM) images of SL, SL/Cel, and SL /Amy. The samples were aqueous solutions with a concentration of 100 g/L after aging for 24 h.

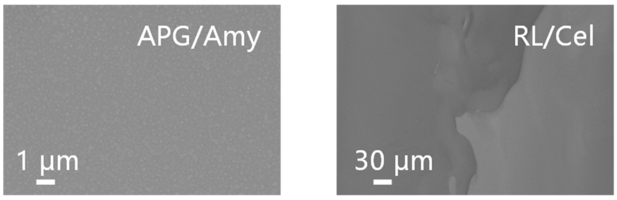

Figure S10. Scanning electron microscopy (SEM) images of APG/Amy, and RL/Cel. The samples were aqueous solutions with a concentration of 100 g/L after aging for 24 h.
